# C239S Mutation in the β-Tubulin of *Phytophthora sojae* Confers Resistance to Zoxamide

**DOI:** 10.3389/fmicb.2016.00762

**Published:** 2016-05-20

**Authors:** Meng Cai, Jianqiang Miao, Xi Song, Dong Lin, Yang Bi, Lei Chen, Xili Liu, Brett M. Tyler

**Affiliations:** ^1^Department of Plant Pathology, China Agricultural UniversityBeijing, China; ^2^Department of Botany and Plant Pathology, Oregon State University, CorvallisOR, USA; ^3^Plant Science and Technology College, Beijing University of AgricultureBeijing, China; ^4^College of Forestry, Beijing Forestry UniversityBeijing, China

**Keywords:** zoxamide, β-tubulin inhibitor, oomycetes, resistance risk, molecular mechanism of resistance, C239S

## Abstract

Zoxamide is the sole β-tubulin inhibitor registered for the control of oomycete pathogens. The current study investigated the activity of zoxamide against *Phytophthora sojae* and baseline sensitivity was established with a mean EC_50_ of 0.048 μg/ml. The data is critical for monitoring changes in zoxamide-sensitivity in the field. Three stable resistant mutants with a high resistance level were obtained by selection on zoxamide amended media. Although the development of resistance occurred at a low frequency, there were no apparent fitness penalty in the acquired mutants in terms of growth rate, sporulation, germination and pathogenicity. Based on the biological profiles and low mutagenesis rate, the resistance risk of *P. sojae* to zoxamide can be estimated as low to medium. Further investigation revealed all the zoxamide-resistant mutants had a point mutation of C239S in their β-tubulin. Zoxamide also exhibited high activity against most species from the genus *Pythium* in which only *Pythium aphanidermatum* was found naturally resistant to zoxamide and harboring the natural point mutation S239 in the β-tubulin. Back-transformation in *P. sojae* with the mutated allele (S239) confirmed the C239S mutation can induce resistance to zoxamide, and the resistance level was positively related to the expression level of the mutated gene. In contrast, the overexpression of the wild type gene was unable to cause zoxamide resistance. It is the first report on the resistance molecular mechanism of zoxamide in oomycetes. Based on our study, C239 is supposed to be a key target site of zoxamide, which distinguishes zoxamide from benzimidazoles and accounts for its low resistance risk. The result can provide advice on the design of new β-tubulin inhibitors in future.

## Introduction

Many oomycetes are destructive pathogens that cause damages to a wide range of agriculturally important plants and animals and also to species associated with forestry and natural ecosystems (Phillips et al., 2008; Kamoun et al., 2015). Among the plant-pathogenic oomycetes, species of the genus *Phytophthora* are highly disruptive to most dicotyledonous plants (Erwin and Ribeiro, 1996) and causes huge losses to production every year (Fry and Goodwin, 1997; Rizzo et al., 2005; Tyler, 2007; Haverkort et al., 2008). More specifically, *Phytophthora sojae* has been shown to be one of the most important causal agents of root and stem rot diseases in soybeans, causing annual losses of $200–300 million in the US alone and global losses estimated to be around $1–2 billion (Tyler, 2007). Despite the development of resistance varieties and the application of chemical controls, the management of oomycete diseases remains a significant challenge for agriculture. Most resistant cultivars rely on major resistance genes, such as the *R* genes (Resistance genes), which can be easily overcome by the pathogens (Fry, 2008). Furthermore, none of the currently utilized *R* genes provide effective control against all pathogen races. The process of discovering new resistance genes is difficult and the breeding of resistant cultivars also has its limitations. Consequently, the development of effective methods of chemical control seems to be a more viable solution (Whisson et al., 2011). However, only a very few compounds are available for the control of oomycetes because these plant pathogens are phylogenetically distinct from true fungi (Beakes et al., 2012) that can be controlled by many commercial fungicides. According to the Fungicide Resistance Action Committee (FRAC), the number of fungicides which can be used for oomycete control is very limited and the development of fungicide resistance could represent a major risk for the management of oomycete diseases.

Zoxamide is a microtubule inhibitor registered for the control of oomycete pathogens in a range of crops including potatoes, vines, and other vegetables (Egan et al., 1998). Similar to benzimidazole fungicides, zoxamide also has activity against a range of true fungi including *Botrytis cinerea, Monilinia fructicola, Venturia inaequalis, Mycosphaerella fijiensis*, and *Cercospora beticola* (Egan et al., 1998). Indeed, it is reported that the two types of fungicides share the same mode of action by binding to the β-subunit of microtubules to inhibit tubulin polymerization and arrest nuclear division (Young and Slawecki, 2001). Furthermore, *in vitro* tests have shown that zoxamide can compete with colchicine for binding sites on the cysteine residue 239 of the β-tubulin protein (Young, 2007). The resistance to the conventional benzimidazoles, including benomyl and carbendazim was developed rapidly after their introduction (Malathrakis, 1979). However, the resistance or reduced sensitivity to zoxamide has rarely been reported since its commercialization in 2001 (Young et al., 2001; Bi et al., 2011, 2014; Malandrakis et al., 2011). Furthermore, attempts to obtain zoxamide-resistant *Phytophthora* isolates via chemical mutagenesis, UV irradiation and selective adaptation have been largely unsuccessful (Young et al., 2001; Young, 2007). To date, there have been just two reports of zoxamide resistance in oomycete pathogens. The first resistance was acquired with a low frequency in *P. capsici* by exposing *P. capsici* zoospores and mycelial cultures to UV irradiation and selection on zoxamide amended media (Bi et al., 2011). The second resistance was acquired from *Pythium sylvaticum* by repeated zoxamide treatments in the laboratory (Martinez et al., 2005). Although obvious resistance was detected in the mutants of both species, the mechanism of resistance in oomycetes remains unknown (Young et al., 2001; Martinez et al., 2005; Bi et al., 2011, 2014). On the contrary, it is reported in true fungi that the mutation of M233I in the β-tubulin protein conferred the zoxamide resistance in *B. cinerea* (Cai et al., 2015).

The current study was initiated to determine the baseline sensitivity of wild-type *P. sojae* isolates to zoxamide and assess the frequency of resistance in response to *in vitro* selection. Furthermore, we have investigated the molecular mechanism of resistance to zoxamide in *P. sojae* by characterizing mutations in the β*-tubulin* gene and back transformed the mutated genes to validate the mechanism of resistance in *P. sojae*.

## Materials and Methods

### Isolates and Culture Conditions

The wild-type *P. sojae* isolates used in the study are listed in Supplementary Table S1. All the isolates were cultured in darkness on V8 plates (200 ml V8 juice, 1g CaCO_3_, 15 g agar, distilled water to 1 l) at 25°C. For long-term storage, the isolates were maintained in 5 ml plastic tubes containing V8 medium slants under mineral oil at 18°C or frozen in liquid nitrogen (Dorrance et al., 2008).

### Fungicide Sensitivity Assays

Technical grade zoxamide (97.5% a.i.; Gowan Company, LLC, Yuma, AZ, USA) was dissolved in dimethyl sulfoxide (DMSO) to prepare a stock solution (5 × 10^4^ μg/ml), which was kept in darkness at -20°C until required. The final concentration of the solvent (DMSO) in the tested media was limited to 0.1% (v/v), which was the level causing no inhibition of mycelia growth in preliminary tests. The same concentration of DMSO was used as a control throughout this study.

The sensitivity tests were performed on V8 plates amended with zoxamide at the following concentrations 0, 0.02, 0.03, 0.04, 0.05, 0.06, 0.07, or 0.1 μg/ml. Mycelia plugs (5 mm in diameter) were excised from the growing edges of 5-day-old *P. sojae* colonies and transferred to the test V8 plates. Three replicate plates were used for each of the different fungicide concentrations. The effect of the fungicide on mycelia growth was determined by measuring the colony diameters after 4 days of incubation in darkness at 25°C. The data collected was subjected to linear regression according to the method described in a previous study (Lu et al., 2010), and the effective concentration for 50% inhibition (EC_50_) was calculated from the dose response curves after probit analysis.

### Generation of Zoxamide-Resistant *P. sojae* Mutants

Ten wild-type *P. sojae* isolates (Ps6, Ps13, Ps15, AH5, AH7, CX16, SH6, SH8, PsJMS1, and PsJMS2) were randomly selected for the screening experiments to produce zoxamide-resistant mutants. Mycelial agar plugs were cut from 5-day-old colonies and transferred to V8 plates containing 1 μg/ml of zoxamide (the concentration at which the growth of all the wild-type isolates was inhibited). After dark-incubation at 25°C for 15–30 days, the area containing the fastest-growing portion of the each colony was transferred to fresh V8 plates amended with increasing concentrations of zoxamide (5, 10, and 50 μg/ml). Finally, the surviving colonies were sub-cultured for three successive times on V8 plates containing 50 μg/ml of zoxamide to stabilize the resistance. The EC_50_ values of the zoxamide-resistant mutants were then estimated by measuring mycelial growth on V8 plates containing 1, 3, 5, 10, 25, and 50 μg/ml zoxamide. The resistance factor (RF) for each mutant was calculated by dividing the EC_50_ of the corresponding wild-type parental isolate by the EC_50_ of the mutant.

### Biological Characterization of Zoxamide-Resistant Mutants

#### Resistance Stability of Zoxamide-Resistant Mutants

The zoxamide-resistant mutants and their corresponding parents were subjected to 10 successive transfers on fungicide-free V8 plates. At each transfer, the mycelial plugs excised from the edge of 5-day-old colonies were placed on a new fungicide-free V8 plate (one plug per plate), with three replicate plates per isolate. The EC_50_ values (or inhibition ratio on 50 μg/ml of zoxamide) were determined after the 1st, 5th, and 10th transfers. The entire experiment was conducted twice for each selected mutant/parental isolate pair.

#### Mycelial Growth Rate at Series of Temperatures

Responses of the mutants and parental isolates to varying temperatures were compared by culturing them on V8 plates in darkness at a range of different temperatures: 4, 12, 20, 25, 28, and 37°C. After 5 days of incubation, the colony diameter was measured. Each combination of isolate or mutant and temperature was represented by three replicate plates, and the experiment was conducted twice.

#### Sporulation and Cyst Germination

The zoospore production of the zoxamide-resistant mutants and their parental isolates was compared after 5 days of culture on V8 plates. The production of zoospores was induced by repeatedly washing the plates with distilled water as described by Morris and Ward (1992). Each isolate or mutant was represented by 10 replicate plates. Sporulation was quantified with a hemacytometer and expressed as the number of zoospores per square centimeter of culture. The germination of the cystospores was determined by microscopy after overnight of incubation on V8 agar at 25°C in the dark (Bi et al., 2011). The entire experiments were conducted twice.

#### Virulence Assay

Virulence was determined by inoculating detached soybean leaves (cv. Williams) with 10 μl of zoospore suspension containing 10^5^ zoospores/ml. Newly expanded trifoliate leaves from 9 to 12 day-old seedlings were used for the assays, and the lesion area on each leaf was measured after 2–3 days of dark-incubation at 25°C. Ten leaves were used for each isolate or mutant and the entire experiments were performed twice.

### Cross Resistance Assay

The zoxamide-resistant mutants were exposed to five fungicides belonging to different chemical groups (chlorothalonil, azoxystrobin, cymoxanil, metalaxyl, and flumorph), all of which are commonly used in China^1^. The EC_50_ values for each mutant/fungicide combination were determined by using the mycelia growth inhibition method described above. The concentrations used for each fungicide are listed in Supplementary Table S2.

### Nucleic Acid Manipulations

Total DNA was extracted from the mycelia of *P. sojae* according to the protocol of Chen et al. (2012). The PCR reaction was performed by using Takara PrimeSTAR HS DNA Polymerase (Takara Biotechnology Co. Ltd, Dalian, China) according to the manufacturer’s protocol, and the primers were listed in Supplementary Table S3. The PCR products were purified by using the EasyPure^®^ PCR Purification Kit (TransGen Biotech Co., Beijing, China) and sequenced by Beijing Sunbiotech Co., Ltd. The sequence data was analyzed by using DNAMAN (Version 8) and Chromas (Version 2.23) software.

The total RNA was extracted from lyophilized mycelial tissue by using the SV Total RNA Isolation System (Promega Biotechnology Co. Ltd, Beijing, China). First-strand cDNA was then synthesized by using the PrimeScript^TM^ RT Reagent Kit with gDNA Eraser (Perfect Real Time) (Takara Biotechnology Co. Ltd, Dalian, China). For gene expression analysis, the SYBR green real-time RT-PCR was then performed according to the manufacturer’s protocol [Takara, SYBR^®^ Premix Ex Taq^TM^ (Tli RNaseH Plus), Bulk]. The used primers were described in Supplementary Table S3.

### Transformation of *P. sojae*

The transformation vector was prepared by cloning the β*-tubulin* genes from the mutant and the wild-type isolates of *P. sojae* into the *StuI* site in plasmid pYF2 (Fang and Tyler, 2016). The enzyme digestions and ligations (New England BioLabs Inc) were performed according to the manufacturer’s protocol. The orientation and integrity of the insert were confirmed by DNA sequencing.

The transformation of *P. sojae* was accomplished by following the protocol described in Fang and Tyler (2016). The transformants were initially screened in pea broth medium amended with 30 μg/ml geneticin (AG Scientific) and then on V8 agar plates containing 50 μg/ml geneticin. The sensitivity of the isolated transformants to zoxamide was measured via the sensitivity assay as described above.

## Results

### Baseline Sensitivity of *P. sojae* Field Isolates to Zoxamide

**FIGURE 1:**
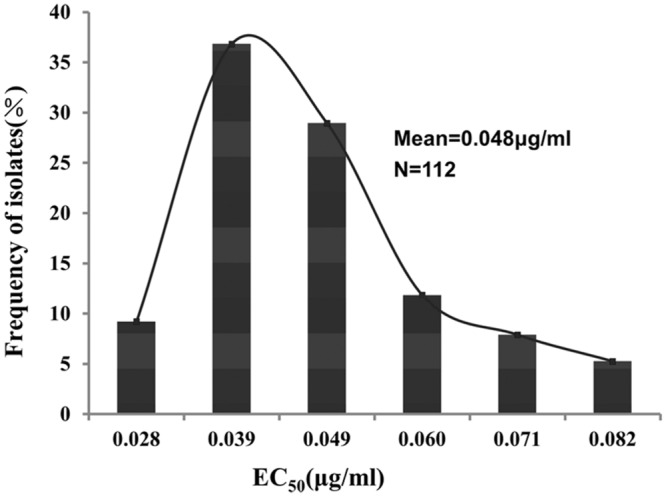
**Frequency distribution of zoxamide EC_50_ values (effective concentrations for 50% inhibition of mycelial growth) for 112 field isolates of *P. sojae***.

The EC_50_ values for zoxamide to inhibit mycelial growth of 112 *P. sojae* field isolates ranged from 0.023 to 0.086 μg/ml (mean = 0.048 μg/ml; Supplementary Table S1). The difference between the maximum and the minimum EC_50_ was 3.76 fold. The frequency distribution of the EC_50_ values from the isolates is illustrated in **Figure 1**, which exhibited a unimodal curve with a positive skew (**Figure 1**).

### Generation of Zoxamide-Resistant Mutants

Three mutants, designated as RZ8-1, RZ11-1, and RZ14-1, were successfully obtained at a low frequency from mycelial adaptation of the wild-type isolate PsJMS2 on zoxamide-amended medium (**Table 1**). The precise EC_50_ values of the resistant mutants could not be determined due to the solubility of zoxamide. The three mutants showed a growth rate suppressed only by less than 10% at a zoxamide concentration of 50 μg/ml, indicating that their EC_50_ values could be far above the tested concentration, and therefore the corresponding RF (RF = EC_50_ of the resistant mutant/EC_50_ of the parent isolate) values were above 1000 (**Table 1**). No zoxamide-resistant mutants were derived from the other nine *P. sojae* isolates Ps6, Ps13, Ps15, AH5, AH7, CX16, SH6, SH8, or PsJMS1.

**Table 1:** Stability of resistance to zoxamide in three *P. sojae* mutants.

Isolate	EC_50_	RF^a^	Inhibition ratio on 50 μg/ml zoxamide		
			1st %	5th %	10th %
RZ11-1	>50 μg/ml	>1000	8	7	3
RZ14-1	>50 μg/ml	>1000	2	9	9
RZ8-1	>50 μg/ml	>1000	6	10	7
PsJMS2	0.04 μg/ml	–	100	100	100

*^a^RF, resistance factor; RF, EC_50_ of the resistant mutant/EC_50_ of the parent isolate (PsJMS2). The RF and EC_50_ of the parent and mutants remained unchanged across the three generations.*

### Stability of Resistance

The stability of the zoxamide-resistant mutants, RZ8-1, RZ11-1, and RZ14-1, was evaluated after ten successive subcultures on fresh fungicide-free media. The inhibition ratio of 50 μg/ml zoxamide to the 5th and 10th generation colonies remained below 10%, which was the same level as for the first generation (**Table 1**). These results indicate that the resistance was stable in the three mutants.

### Mycelial Growth, Sporulation, Cyst Germination, and Virulence

The mycelial growth, sporulation, cystospore germination and virulence of the three mutants were compared to the parental isolate PsJMS2 and two other wild-type isolates (AH1204, Ps6) which were selected randomly. Although the mycelial growth of RZ14-1 was significantly reduced (*p* < 0.05) compared with the parental isolate, it was comparable to the other wild-type isolates AH1204 and Ps6 (**Table 2**). RZ11-1 grew fastest among all the isolates while RZ8-1 grew slowest (*p* < 0.05) (**Table 2**). With the exception of RZ14-1, which produced the most zoospores, there were no significant differences (*p* < 0.05) between the zoospore production of the resistant mutants and wild-type isolates (**Table 2**). Furthermore, all the isolates tested had consistently high rates of cystospore germination, which were all in excess of 97% (**Table 2**). RZ11-1 and PsJMS2 produced the largest lesion size, which did not significantly differ from each other (*p* < 0.05). Although the virulence of RZ8-1 and RZ14-1 was reduced compared with the parental isolate PsJMS2, they were still higher than either of the other two wild-type isolates, and the difference was not significant (*p* < 0.05) (**Table 2**).

**Table 2:** Comparison of fitness parameters for zoxamide-resistant mutants of *P. sojae* and several sensitive wild-type.

Isolate	Colony diameter (mm) at 108 h	Zoospore production *in vitro* (× 10^5^/cm^2^)	Germination rate (%)	Lesion area on detached leaves (cm^2^)
PsJMS2	(50.0 ± 0.6)^b^	(2.3 ± 0.3)b	(98.0 ± 0.5)ab	(7.7 ± 0.9)a
AH1204	(46.7 ± 0.6)^c^	(2.7 ± 0.3)b	(98.0 ± 0.7)ab	(2.6 ± 1.4)c
Ps6	(47.7 ± 0.7)^c^	(4.0 ± 0.6)ab	(99.7 ± 0.2)a	(2.3 ± 0.6)c
RZ11-1	(55.2 ± 0.3)^a^	(4.0 ± 0.6)ab	(98.0 ± 0.5)ab	(7.34 ± 0.9)ab
RZ14-1	(46.7 ± 0.4)^c^	(5.0 ± 0.6)a	(99.2 ± 0.6)a	(4.01 ± 1.5)abc
RZ8-1	(44.3 ± 0.7)^d^	(3.0 ± 0.6)ab	(97.3 ± 0.6)b	(3.04 ± 1.3)bc

*For the wild-type isolates and the resistant mutants, means followed by same letters are not significantly different according to Fisher’s least significance difference (a = 0.05).*

### Zoxamide-Resistant Mutants Show No Cross Resistance to Other Fungicides

The activities of five conventional fungicides (chlorothalonil, azoxystrobin, cymoxanil, metalaxyl and flumorph) belonging to different chemical groups other than β-tubulin inhibitors were tested for cross resistance with zoxamide. All the evaluated isolates, regardless of the resistance to zoxamide, were sensitive to the five fungicides tested. The corresponding spearman’s rho values confirmed that there was no cross resistance between zoxamide and the other common fungicides (**Table 3**).

**Table 3:** The cross-resistance between zoxamide and five conventional fungicides.

	EC_50_ (μg/ml)					
	Zoxamide	Chlorothalonil	Azoxystrobin	Cymoxanil	Metalaxyl	Flumorph
PsJMS2	0.05	3.74	0.47	0.25	0.06	0.55
JN4	0.05	5.64	0.72	0.58	0.66	0.51
JSJ-07-2	0.04	5.77	0.65	0.31	0.06	0.31
AH1303	0.04	6.33	0.58	0.67	0.41	0.39
FJ3	0.04	5.89	0.42	0.73	0.24	0.37
SJD-07-3	0.05	7.85	0.79	0.82	0.23	0.49
AH1204	0.04	7.00	0.66	0.21	0.31	0.45
Ps6	0.04	6.54	0.44	0.68	0.63	0.30
RZ11-1	>50	5.62	0.56	0.23	0.08	0.45
RZ14-1	>50	4.89	0.77	0.19	0.30	0.44
RZ8-1	>50	4.89	0.46	0.23	0.29	0.41

### Zoxamide-Resistant Mutants Carry a Mutation of C239S in β-Tubulin

**FIGURE 2:**
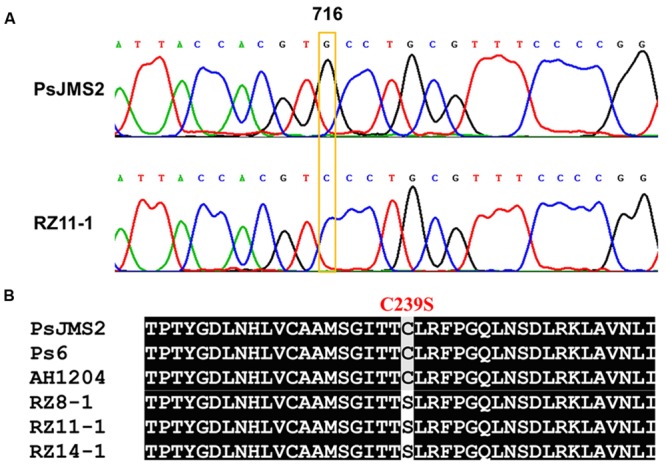
**(A)** Sequencing chromatograms of the β*-tubulin* gene from zoxamide-resistant mutant RZ11-1 and the sensitive parental isolate PsJMS2. **(B)** Molecular characterization of the β-tubulin amino acid sequence from the three *P. sojae* mutants, their parental isolate PsJMS2, and two other field isolates, Ps6 and AH1204. Eight other sensitive field isolates had the same sequence as PsJMS2.

The *β-tubulin* gene was cloned and sequenced from zoxamide-resistant and -sensitive isolates, respectively. Multiple sequence alignment of the β*-tubulin* genes revealed a G-to -C transversion at the nucleotide position 716 in the zoxamide-resistant mutants (**Figure 2A**). The point mutation G716C resulted in the substitution of serine for the conserved cysteine residue at the amino acid position 239 (**Figure 2B**). In addition, the investigation revealed that the zoxamide-resistant mutants produced only a single chromatogram-peak at the nucleotide position 716 (**Figure 2A**), which indicated that the C239S mutation was homozygous in the zoxamide-resistant mutants in consideration of the diploid characteristic of oomycetes.

### S239 Naturally Occurs in *Pythium aphanidermatum* Field Isolates Which Are Inherently Resistant to Zoxamide

Zoxamide also exhibits a high level of activity against most species from the genus *Pythium* such as *Pythium ultimum, Pythium sulcatum, Pythium sylvaticum*, and *Pythium macrosporum* (Martinez et al., 2005). *Pythium aphanidermatum*, the sensitivity of which to zoxmide has not been reported before, was found to be an exception in our study. The EC_50_ values to zoxamide in two *Pythium aphanidermatum* wild-type isolates (*Pythium Aphanidermatum* 1 *and Pythium aphanidermatum* 2) were 50.63 and 51.63 μg/ml, which were more than 500 folds of the EC_50_ values in *P. sojae, P. infestans, P. capsici*, and *Pythium ultimum* (0.05, 0.05, 0.08, and 0.07 μg/ml, respectively, **Table 4**). The result indicates that *Pythium aphanidermatum* is naturally highly resistant to zoxamide.

**Table 4:** Sensitivities of *P. infestans, P. capsici, Pythium ultimum*, and *Pythium aphanidermatum* wild-type isolates zoxamide.

Isolate	EC_50_ to zoxamide
*P. sojae*	0.05 μg/ml
*P. infestans*	0.05 μg/ml
*P. capsici*	0.08 μg/ml
*Pythium ultimum*	0.07 μg/ml
*Pythium aphanidermatum 1*	50.63 μg/ml
*Pythium aphanidermatum 2*	51.63 μg/ml

**FIGURE 3:**
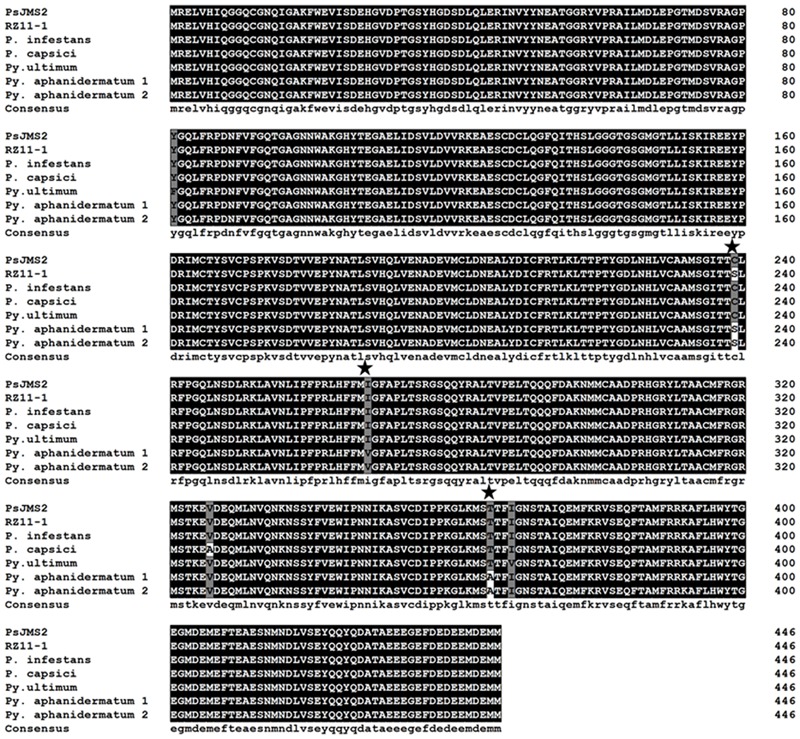
**Multiple alignment of the β-tubulin amino acid sequences in *P. sojae, P. infestans, P. capsici, Pythium ultimum* and *Pythium aphanidermatum* isolates.** PsJMS2 is a wild type *P. sojae* isolate and RZ11-1 is the one of artificial zoxamide-resistant mutants of *P. sojae.* The asterisk (★) indicates the SNPs peculiar to *Pythium aphanidermatum*.

The similarity of the β-tubulin amino acid sequences among *P. sojae, P. infestans, P. capsici, Pythium ultimum* and *Pythium aphanidermatum* was extremely high, which was over 99% (**Figure 3**). Three SNPs, including S239, V268 and A365, were found peculiar to *Pythium aphanidermatum* (**Figure 3**), while the amino acid S239 was found associated with zoxamide resistance in the artificial mutants of *P. sojae* in the above study.

### Back Transformation of *P. sojae* with Wild Type and Mutated *β-Tubulin* Genes

**FIGURE 4:**
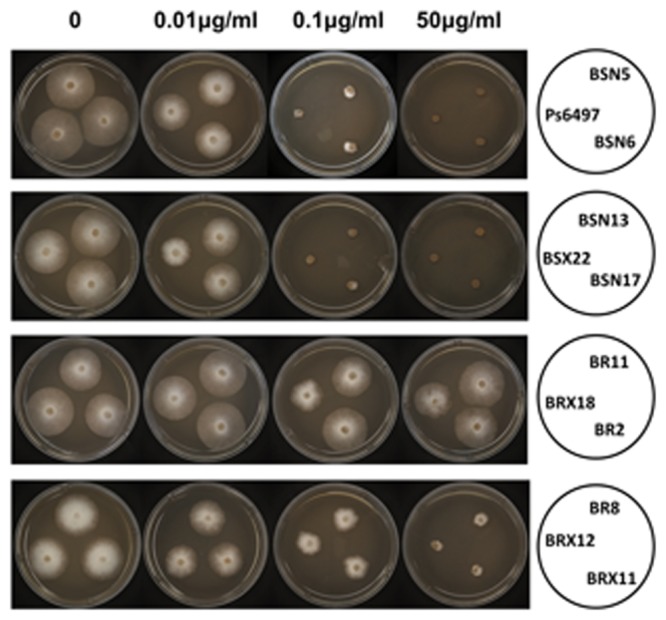
**Mycelia growth of *P. sojae* transformants carrying either C239 or S239 in β-tubulin on zoxamide-amended medium.** P6497 is the sensitive wild-type strain used for transformation. BSN5, BSN6, BSN13, BSX22, and BSN17 carry the C239 allele; BR11, BRX18, BR2, BR8, BRX12, and BRX11 carry the S239 allele.

The mutated *β-tubulin* gene from RZ11-1 (S239) was used to investigate the role of the mutation in zoxamide resistance. The wild-type *β-tubulin* gene from PsJMS2 (C239) was used as a control. The genes were placed under the control of the constitutive promoter HAM34 and introduced into the genome-sequenced isolate P6497 by protoplast transformation. A total of 27 stable transformants were recovered, in which 12 (TrBRX18, TrBR2, TrBR11, TrBR8, TrBRX12, TrBRX11, TrBRN10, TrBRN16, TrBRN14, TrBRN12, TrBRN15 and TrBRX9) contained S239 β-tubulin allele, and the other 15 (TrBSN5, TrBSN6, TrBSN13, TrBSN17, TrBSX22, TrBSX9, TrBSN1, TrBSX19, TrBSX20, TrBSX21, TrBSN8, TrBSX1, TrBSX2, TrBSX12 and TrBS4) contained C239 β-tubulin allele. Since the baseline sensitivity to zoxamide of *P. sojae* is 0.05 μg/ml, we used the concentration 1 μg/ml of zoxamide on which the wild-type isolates cannot grow, to detect whether the transformants are resistant to zoxamide or not. The result showed the above 12 transformants containing S239 β-tubulin allele can grow on 1 μg/ml of zoxamide; the above 15 transformants containing C239 β-tubulin allele cannot grow on 1 μg/ml of zoxamide. Seven of the transformants, TrBR2, TrBR8, TrBRX18 and TrBSN5, TrBSN6, TrBSN13, TrBSX22 were selected for further characterization. Sensitivity assays revealed that the transformants TrBR2, TrBR8, TrBRX18 carrying the S239 allele exhibited varied levels of resistance to zoxamide (**Table 5**, **Figure 4**), while none of the transformants with the wild-type allele TrBSN5, TrBSN6, TrBSN13, TrBSX22 exhibited any resistance, which were unable to grow on V8 plates containing 0.1 μg/ml zoxamide (**Table 5**, **Figure 4**).

**Table 5:** Sensitivity of *P. sojae* transformants containing either the C239 or S239 β-tubulin allele to zoxamide and corresponding levels of gene expression.

Strain	EC_50_ (μg/ml)	RF^a^	Expression Level		Ratio (Transgene β-tubulin/Endogenous β- tubulin)
			Endogenous β- tubulin	Total β- tubulin	
P6497	0.02	–	1.00	1.00	–
TrBSN5	0.03	1.26	0.84	24.2	27.9
TrBSN6	0.03	1.20	0.36	39.6	108.0
TrBSN13	0.02	0.70	0.81	8.52	9.49
TrBSX22	0.02	0.83	0.54	4.83	7.96
TrBRX18	>50	>2220	0.63	1.93	21.7
TrBR2	>50	>2220	0.54	7.47	12.8
TrBR8	0.11	5.04	1.94	44.2	2.04

*^a^RF, resistance factor; RF, EC_*50*_ of the transformants/EC_*50*_ of the parental wild-type isolate (P6497).*

### Sensitivity of Transformants to Zoxamide and the *β-Tubulin* Expression Level

Two of the transformants containing the S239 allele, TrBRX18 and TrBR2, exhibited a high level of resistance to zoxamide (RF > 1000, **Table 5**), which were consistent with the original mutants RZ11-1, RZ14-1, and RZ8-1; while for TrBR8, it exhibited a lower level of resistance with a RF only 5 fold higher than that of the sensitive wild-type isolate P6497 (**Table 5**). In contrast, all of the transformants containing the wild-type allele (TrBSN5, TrBSN6, TrBSN13, and TrBSX22) were sensitive to zoxamide, which had EC_50_ values similar to the sensitive wild-type isolate P6497 (**Table 5**).

The transcript level of the introduced allele, relative to the background expression of the endogenous *β-tubulin* allele, was investigated via qRT-PCR. The results revealed that the introduced alleles, regardless of S239 or C239, were all expressed at a higher level than the endogenous *β-tubulin* allele (**Table 5**). The resistance level of the transformants with S239-allele (TrBRX18, TrBR2, and TrBR8), was found to be positively related to the expression level of the mutated gene (**Table 5**). In contrast, the overexpression of the wild type *β-tubulin* gene was unable to cause zoxamide resistance in the transformants with C239-allele (TrBSN5, TrBSN6, TrBSN13, and TrBSX22).

## Discussion

Although benzimidazoles, a group of conventional fungicides with a long history of application, share a similar mode of action with zoxamide, they have been only effective in the control of true fungi but not oomycetes on account of the latter’s peculiar physiological characteristics resulting in phylogenetically distance from true fungi (Beakes et al., 2012). Consequently zoxamide is the only β-tubulin inhibitor registered for the control of oomycete diseases (Egan et al., 1998; Young, 2007). However, despite the two groups of fungicides sharing a similar mode of action, it has been noted that in contrast to the benzimidazoles, for which the high risk of resistance has developed in a large range of pathogens worldwide (Malathrakis, 1979; Davidse and Ishii, 1995; Malandrakis et al., 2011), the resistance risk of zoxamide is defined as low by FRAC. Resistant isolates have never been observed in the field and the frequency of resistant mutants is low in laboratory studies (Young et al., 2001; Martinez et al., 2005; Young, 2007; Bi et al., 2011, 2014). Therefore, it has been suggested that the disparity between the resistance risk for these two fungicides results from their specific target sites on the β-tubulin protein.

The current study established baseline sensitivity to zoxamide in the Chinese population of *P. sojae*. The EC_50_ values of 112 isolates ranged from 0.022 to 0.086 μg/ml, which are similar to the level of zoxamide sensitivity in other *Phytophthora* pathogens such as in *P. capsici* (Bi et al., 2011) and *P. cactorum* (Mei et al., 2014). Furthermore, the distribution of the EC_50_ values of the 112 *P. sojae* isolates exhibited a unimodal curve, which indicated that no natural spontaneous mutants had been detected. Consequently, the baseline sensitivity of zoxamide established in the current study can be adopted for future monitoring changes in the zoxamide sensitivity of *P. sojae* populations in field.

In this study, three stable resistant mutants exhibiting a high level of zoxamide resistance (RF > 1000, **Table 1**) were obtained by zoxamide-adaption. Comparison of the biological characteristics of the mutants with the parental isolate and two other wild-type isolates indicated that there was no fitness penalty with regard to the growth rate, the sporulation, the cystospore germination and the virulence. These results suggest that if such a mutation arises in field populations of *P. sojae*, they would not be at a competitive disadvantage against sensitive isolates. However, the rate of mutagenesis was extremely low and was only found to occur in one of 10 parental isolates which were randomly selected for the mutant screening. Taken together, these results indicate that the resistance risk of *P. sojae* to zoxamide should be considered to be low to medium. Given the competitive fitness of the resistant mutants, it is advisable to closely monitor any sensitivity changes to zoxamide in *P. sojae* in case such a resistance will arise and proliferate in field populations. The study also revealed that there is no cross resistance between zoxamide and the five conventional fungicides including chlorothalonil, azoxystrobin, cymoxanil, metalaxyl and flumorph, which indicates that zoxamide could be applied alternately or used in mixtures with the five fungicides in order to delay the development of resistance.

Previous studies have demonstrated that zoxamide and colchicine compete for binding sites on the cysteine at codon 239 of the β-tubulin protein to lead to the arrest of nuclear division (Young, 2007). However, studies of zoxamide resistance in *P. capsici* (Bi et al., 2011, 2014), *P. cactorum* (Mei et al., 2014) and *Pythium sylvaticum* (Martinez et al., 2005) have shown that the zoxamide resistance in these mutants is not caused by mutations in the *β-tubulin* gene or its overexpression. These results indicate that the zoxamide resistance in these oomycete species probably results from non-target-site based mechanisms (Bi et al., 2014; Mei et al., 2014). Bi et al. (2014) suggested that the resistance to zoxamide in *P. capsici* was controlled by two non-target recessive genes, in which resistance would occur when at least one pair of those alleles was homozygous. However, the current study revealed that the resistance observed in *P. sojae* was caused by the substitution of serine for the conserved cysteine residue at position 239 in the β-tubulin protein. And C239 happens to be a reported binding site of zoxamide (Young, 2007). Even though zoxamide exhibits a high level of activity against vast majority of oomycete pathogens, including most species from the genus *Pythium* such as *Pythium ultimum, Pythium sulcatum, Pythium sylvaticum*, and *Pythium macrosporum* (Martinez et al., 2005), a notable exception is *Pythium aphanidermatum*, which is completely unaffected by zoxamide treatment. It is therefore interesting to note that multiple sequence alignment of the amino acid sequences of β-tubulin of several *Phytophthora* and *Pythium* species including *P. sojae* (sensitive to zoxamide), *P. infestans* (sensitive to zoxamide), *P. capsici* (sensitive to zoxamide), *Pythium ultimum* (sensitive to zoxamide) and *Pythium aphanidermatum* (inherent resistant to zoxamide) revealed that although the species share greater than 98% sequence identity, in contrast to all the other species, *Pythium aphanidermatum* alone possesses a serine residue at position 239 instead of the usual cysteine. This result is particularly meaningful given that C239 is highly conserved in the β-tubulin of a broad range of taxa including oomycetes, true fungi, plants and animals (Supplementary Figure S1). Taken together, these results provide compelling evidence that the C239S substitution in the β-tubulin of the *P. sojae* mutants is responsible for the observed zoxamide resistance, and also that C239 is a key target site of zoxamide, which distinguishes zoxamide from benzimidazoles and accounts for its low resistance risk.

The current study utilized back-transformation experiments to validate the hypothesis that the C239S in the β-tubulin of the mutants resulted in zoxamide resistance. All of transformants containing the S239-allele exhibited resistance to zoxamide, and the resistance level was found to be positively related to the expression level of the mutated gene. In contrast, the transformants containing the C239-allele exhibited sensitivity similar to the parental isolate, and the overexpression of the wild type β*-tubulin* gene (C239-allele) was unable to cause zoxamide resistance. Furthermore, it was also noted that the sensitive protein (the endogenous β-tubulin allele) was still produced in the resistant transformants, although at a low level of expression. Therefore, it is likely that the zoxamide-resistance observed in *P. sojae* would exhibit dominant.

## Author Contributions

MC wrote the main manuscript text and did most parts of the work; JM prepared **Tables 1** and **4**; XS and DL prepared **Table 3**; YB and LC prepared **Figure 1**; BT supervised the transformation work of the study; XL supervised the whole study. All authors reviewed the manuscript.

## Conflict of Interest Statement

The authors declare that the research was conducted in the absence of any commercial or financial relationships that could be construed as a potential conflict of interest.

## Footnotes

1 http://www.chinapesticide.gov.cn/

## References

[B1] Beakes G. W. Glockling S. L. Sekimoto S. (2012). The evolutionary phylogeny of the oomycete “fungi”. *Protoplasma* 249 3–19. 10.1007/s00709-011-0269-2 21424613

[B2] Bi Y. Chen L. Cai M. Zhu S. S. Pang Z. L. Liu X. L. (2014). Two non-target recessive genes confer resistance to the anti-oomycete microtubule inhibitor zoxamide in *Phytophthora capsici*. *PLoS ONE* 9:e89336 10.1371/journal.pone.0089336 PMC3930715 24586697

[B3] Bi Y. Cui X. L. Lu X. H. Cai M. Liu X. L. Hao J. J. (2011). Baseline sensitivity of natural population and resistance of mutants in *Phytophthora capsici* to zoxamide. *Phytopathology* 101 1104–1111. 10.1094/PHYTO-01-11-0010 21692644

[B4] Cai M. Lin D. Chen L. Bi Y. Xiao L. Liu X.-L. (2015). M233I mutation in the β-Tubulin of *Botrytis cinerea* confers resistance to zoxamide. *Sci. Rep.* 5:16881 10.1038/srep16881 PMC4657022 26596626

[B5] Chen L. Zhu S. Lu X. Pang Z. Cai M. Liu X. (2012). Assessing the risk that *Phytophthora melonis* can develop a point mutation (V1109L) in CesA3 conferring resistance to carboxylic acid amide fungicides. *PLoS ONE* 7:e42069 10.1371/journal.pone.0042069 PMC3407118 22848705

[B6] Davidse L. C. Ishii H. (1995). “Biochemical and molecular aspects of the mechanisms of action of benzimidazoles, N-phenylcarbamates and N-phenylformamidoximes and the mechanisms of resistance to these compounds in fungi,” in *Modern Selective Fungicides* ed. Lyr H. (New York, NY: Fischer Verlag).

[B7] Dorrance A. E. Berry S. A. Anderson T. R. Meharg C. (2008). Isolation, storage, pathotype characterization, and evaluation of resistance for *Phytophthora* sojae in soybean. *Plant Health Prog.* 10:1094.

[B8] Egan A. R. Michelotti E. L. Young D. H. Wilson W. J. Mattioda H. (1998). “RH-7281: a novel fungicide for control of downy mildew and late blight,” in *Proceedings of Brighton Crop Protection Conference-Pests and Diseases* Farnham: The British Crop Protection Council 335–342.

[B9] Erwin D. C. Ribeiro O. K. (1996). *Phytophthora Diseases Worldwide.* St. Paul, MN: American Phytopathological Society (APS Press).

[B10] Fang Y. Tyler B. M. (2016). Efficient disruption and replacement of an effector gene in the oomycete *Phytophthora* sojae using CRISPR/Cas9. *Mol. Plant Pathol.* 17 127–139. 10.1111/mpp.12318 26507366 PMC6638440

[B11] Fry W. (2008). *Phytophthora infestans*: the plant (and R gene) destroyer. *Mol. Plant Pathol.* 9 385–402. 10.1111/j.1364-3703.2007.00465.x 18705878 PMC6640234

[B12] Fry W. E. Goodwin S. B. (1997). Resurgence of the Irish potato famine fungus. *Bioscience* 47 363–371. 10.2307/1313151

[B13] Haverkort A. Boonekamp P. Hutten R. Jacobsen E. Lotz L. Kessel G. (2008). Societal costs of late blight in potato and prospects of durable resistance through cisgenic modification. *Potato Res.* 51 47–57. 10.1007/s11540-008-9089-y

[B14] Kamoun S. Furzer O. Jones J. D. Judelson H. S. Ali G. S. Dalio R. J. (2015). The top 10 oomycete pathogens in molecular plant pathology. *Mol. Plant Pathol.* 16 10.1111/mpp.12190 PMC6638381 25178392

[B15] Lu X. H. Zhu S. S. Bi Y. Liu X. L. Hao J. J. (2010). Baseline sensitivity and resistance-risk assessment of *Phytophthora capsici* to iprovalicarb. *Phytopathology* 100 1162–1168. 10.1094/PHYTO-12-09-0351 20932164

[B16] Malandrakis A. Markoglou A. Ziogas B. (2011). Molecular characterization of benzimidazole-resistant *B. cinerea* field isolates with reduced or enhanced sensitivity to zoxamide and diethofencarb. *Pestic. Biochem. Physiol.* 99 118–124. 10.1016/j.pestbp.2010.11.008

[B17] Malathrakis N. E. (1979). Studies on gray mold (*Botrytis cinerea*) of vegetables grown under plastics. *Phytopathol. Mediterr.* 19:70.

[B18] Martinez C. Lévesque C. A. Bélanger R. R. Tweddell R. J. (2005). Evaluation of fungicides for the control of carrot cavity spot. *Pest. Manag. Sci.* 61 767–771. 10.1002/ps.1055 15880371

[B19] Mei X. Yang M. Ding X. Bi Y. Chen L. Deng W. (2014). Proteomic analysis of zoxamide-induced changes in *Phytophthora cactorum*. *Pesticide Biochem. Physiol.* 113 31–39. 10.1016/j.pestbp.2014.06.004 25052524

[B20] Morris P. F. Ward E. (1992). Chemoattraction of zoospores of the soybean pathogen, *Phytophthora sojae*, by isoflavones. *Physiol. Mol. Plant Pathol.* 40 17–22. 10.1016/0885-5765(92)90067-6

[B21] Phillips A. J. Anderson V. L. Robertson E. J. Secombes C. J. van West P. (2008). New insights into animal pathogenic oomycetes. *Trends Microbiol.* 16 13–19. 10.1016/j.tim.2007.10.013 18096392

[B22] Rizzo D. M. Garbelotto M. Hansen E. M. (2005). *Phytophthora* ramorum: integrative research and management of an emerging pathogen in California and Oregon forests. *Annu. Rev. Phytopathol.* 43 309–335. 10.1146/annurev.phyto.42.040803.140418 16078887

[B23] Tyler B. M. (2007). *Phytophthora* sojae: root rot pathogen of soybean and model oomycete. *Mol. Plant Pathol.* 8 1–8. 10.1111/j.1364-3703.2006.00373.x 20507474

[B24] Whisson S. Fonné-Pfister R. Csukai M. Dehne H. Deising H. Gisi U. (2011). “Molecular approaches to elucidate pathways and sites of’fungicide’resistance in oomycetes,” in *Proceedings of the 16th International Reinhardsbrunn Symposium, Modern Fungicides and Antifungal Compounds VI* (Friedrichroda: Deutsche Phytomedizinische Gesellschaft eV Selbstverlag) 91–102.

[B25] Young D. H. (2007). Zoxamide, an antitubulin fungicide for control of oomycete pathogens. *Mod. Crop Prot. Compd.* 2:3.

[B26] Young D. H. Slawecki R. A. (2001). Mode of action of zoxamide (RH-7281), a new Oomycete fungicide. *Pestic. Biochem. Physiol.* 69 100–111. 10.1006/pest.2000.2529

[B27] Young D. H. Spiewak S. L. Slawecki R. A. (2001). Laboratory studies to assess the risk of development of resistance to zoxamide. *Pest Manag. Sci.* 57 1081–1087. 10.1002/ps.399 11721527

