# Supplementary material for: C239S Mutation in the β-Tubulin of Phytophthora sojae Confers Resistance to Zoxamide
Source: Front Microbiol. 2016 May 20;7:762. doi: 10.3389/fmicb.2016.00762 (PMC4873504; doi:10.3389/fmicb.2016.00762)
Supplement: Supplementary file 1 [file Data_Sheet_1.DOCX]

Supplementary Information

Table S1 Information of the 112 *P. sojae* isolates used in the study.

| **Location** | **Code** | | **Coordinates** | **Number** | **EC_50_ range (μg/ml)** | **Year** |
| --- | --- | --- | --- | --- | --- | --- |
| **Fujian** | **Ps1-8** | | **24.78, 118.08** | **8** | **0.035-0.048** | **2008** |
| **Fujian** | **Ps9-27** | | **24.37, 117.91** | **19** | **0.023-0.024** | **2002** |
| **Fujian** | **Ps28-39** | | **24.33, 117.87** | **12** | **0.039-0.053** | **2004** |
| **Fujian** | **Ps40-49** | | **24.77, 118.08** | **10** | **0.033-0.047** | **2005, 2006, 2007** |
| **Heilongjiang** | **LS-** | | **45.12, 130.70** | **15** | **0.028-0.063** | **2010** |
| **Heilongjiang** | **HLYF-** | | **46.89, 126.08** | **9** | **0.032-0.066** | **2010** |
| **Heilongjiang** | **SH-** | | **46.65, 126.98** | **9** | **0.040-0.057** | **2010** |
| **Heilongjiang** | **CX-** | | **46.03, 126.61** | **8** | **0.063-0.086** | **2010** |
| **Heilongjiang** | **DZT3-, JMS-** | | **44.57, 129.63** | **16** | **0.046-0.067** | **2004, 2010** |
| **Anhui** | **AH-** | | **32.36, 117.38** | **6** | **0.040-0.050** | **2010** |
| **Total** |  |  | | **112** | **0.023-0.086** | **2002-2010** |

Table S2 Concentrations used to determine the sensitivities of field isolates and resistant mutants of *P. sojae* to various fungicides

| **Fungicide** | **Concentrations (μg/ml)** |
| --- | --- |
| **chlorothalonil** | **2.0, 4.0, 6.0, 8.0, 10.0** |
| **azoxystrobin** | **0.20, 0.40, 0.80, 1.0, 1.20** |
| **cymoxanil** | **0.10, 0.30, 0.50, 1.0, 3.0, 5.0** |
| **metalaxyl** | **0.15, 0.30, 0.60, 1.2, 2.4** |
| **flumorph** | **0.20, 0.30, 0.40,0.50, 0.60, 0.80** |

Table S3 Primers used in this study.

| **Primer name** | **Sequence 5'-3'** | **Purpose** |
| --- | --- | --- |
| **Ps beta forward** | **GACGGAGAGCCATACGAA** | **Amplification of the β-tubulin gene in *P. sojae*** |
| **Ps beta reverse** | **AGATGCCGAGCCACTAAC** | **Amplification of the β-tubulin gene in *P. sojae*** |
| **qRTHK-2F** | **CTCCAAGGGCTCGTCCAA** | ***P.sojae* housekeeping gene (rpS5) used as qRT-PCR reference** |
| **qRTHK-2R** | **GCCAGCATCCCTCCAAAG** | ***P.sojae* housekeeping gene (rpS5) used as qRT-PCR reference** |
| **PsACT qRT F** | **ACTGCACCTTCCAGACCATC** | ***P.sojae* actin gene used as qRT-PCR reference** |
| **PsACT qRT R** | **CCACCACCTTGATCTTCATG** | ***P.sojae* actin gene used as qRT-PCR reference** |
| **PsBeta_UTRF** | **GTGAGGGTATGGACGAGATGGA** | **qRT-PCR for validation of the endogenous β-tubulin expression patterns** |
| **PsBeta_UTRR** | **TGAACAAAGGCGGTCAGCAA** | **qRT-PCR for validation of the endogenous β-tubulin expression patterns** |
| **PsBeta_qRTF** | **TACCCGGACCGTATCATGTG** | **qRT-PCR for validation of the total β-tubulin expression patterns** |
| **PsBeta_qRTR** | **TCGTACAGGGCCTCGTTATC** | **qRT-PCR for validation of the total β-tubulin expression patterns** |

**
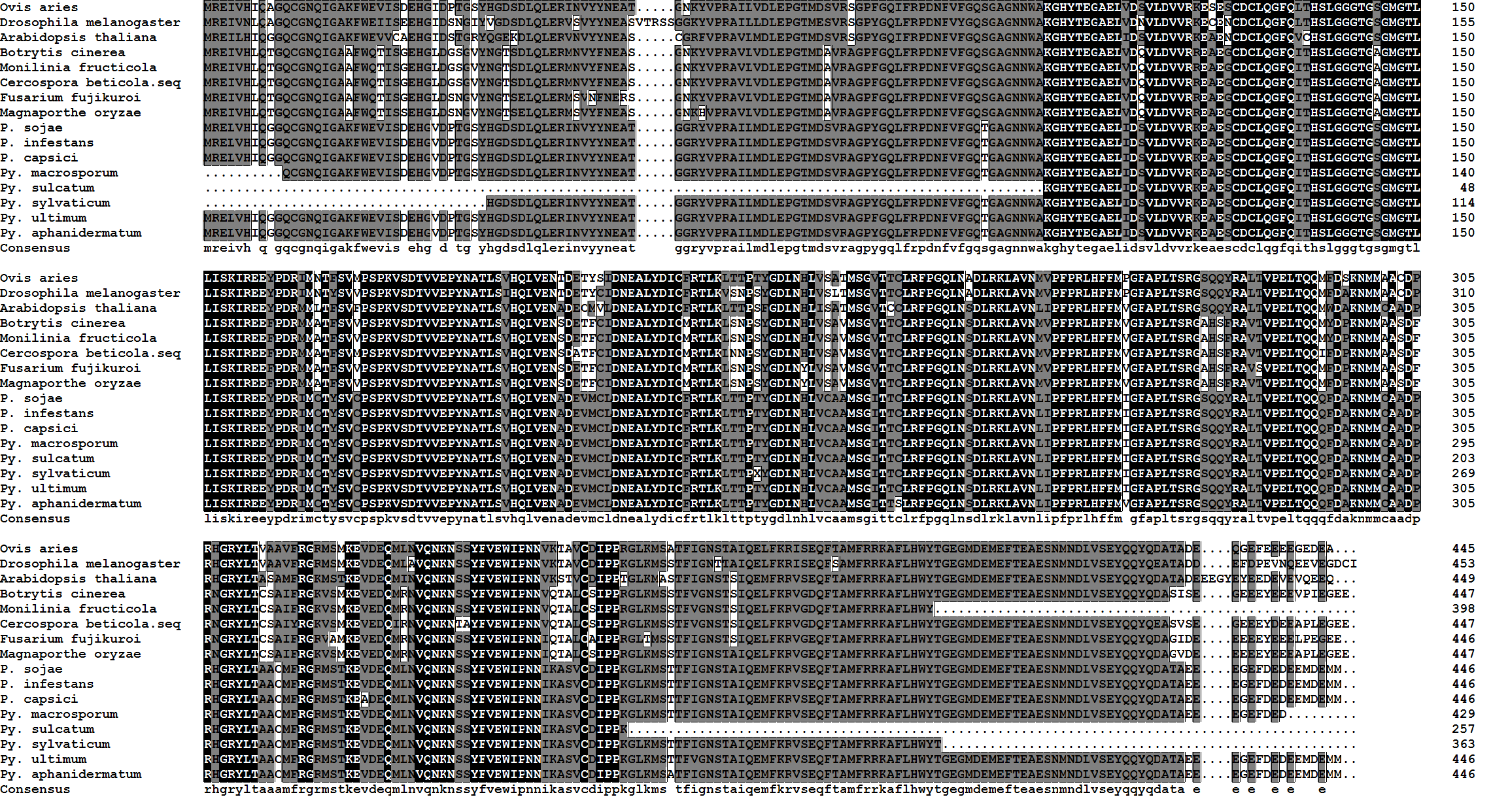
**

**Figure S1 Multiple alignment of the β-tubulin amino acid sequences in *Ovis aries* (PDB: D0VWY9-1), *Drosophila melanogaster* (GenBank: NP_001286835.1), *Arabidopsis thaliana* (GenBank: AAK96884.1), *Botrytis cinerea*, *Monilinia fructicola*, *Cercospora beticola*, *Fusarium fujikuroi*, *Magnaporthe grisea*, *P. sojae*, *P. infestans*, *P. capsici*, *Py. macrosporum* (GenBank: BAJ79093.1), *Py. sulcatum* (GenBank: AJD08746.1), *Py. sylvaticum* (GenBank: ADJ39244.1), *Py. ultimum* and *Py. aphanidermatum*. The red asterisk (★) indicates the amino acid residue at the codon 239.**
